# HALT & REVERSE: Hsf1 activators lower cardiomyocyt damage; towards a novel approach to REVERSE atrial fibrillation

**DOI:** 10.1186/s12967-015-0714-7

**Published:** 2015-11-05

**Authors:** Eva A. H. Lanters, Denise M. S. van Marion, Charles Kik, Herman Steen, Ad J. J. C. Bogers, Maurits A. Allessie, Bianca J. J. M. Brundel, Natasja M. S. de Groot

**Affiliations:** Department of Cardiology, Ba 579, Erasmus Medical Center, ‘s Gravendijkwal 230, 3015 CE Rotterdam, The Netherlands; Department of Clinical Pharmacy and Pharmacology, EB71, University Institute for Drug Exploration (GUIDE), University Medical Center Groningen, University of Groningen, Hanzeplein 1, PO Box 30 001, 9700 RB Groningen, The Netherlands; Department of Cardiothoracic Surgery, Bd 557, Erasmus Medical Center, ‘s Gravendijkwal 230, 3015 CE Rotterdam, The Netherlands; Nyken Therapeutics B.V., L.J. Zielstraweg 2, 9713 GX Groningen, The Netherlands; Department of Physiology, Institute for Cardiovascular Research, VU University Medical Center, Van der Boechorststraat 7, 1081 BT Amsterdam, The Netherlands

**Keywords:** Atrial fibrillation, Mapping, Heat shock proteins, Electropathology

## Abstract

**Background:**

Atrial fibrillation is a progressive arrhythmia, the exact mechanism underlying the progressive nature of recurrent AF episodes is still unknown. Recently, it was found that key players of the protein quality control system of the cardiomyocyte, i.e. Heat Shock Proteins, protect against atrial fibrillation progression by attenuating atrial electrical and structural remodeling (electropathology). HALT & REVERSE aims to investigate the correlation between electropathology, as defined by endo- or epicardial mapping, Heat Shock Protein levels and development or recurrence of atrial fibrillation following pulmonary vein isolation, or electrical cardioversion or cardiothoracic surgery.

**Study design:**

This study is a prospective observational study. Three separate study groups are defined: (1) cardiothoracic surgery, (2) pulmonary vein isolation and (3) electrical cardioversion. An intra-operative high-resolution epicardial (group 1) or endocardial (group 2) mapping procedure of the atria is performed to study atrial electropathology. Blood samples for Heat Shock Protein determination are obtained at baseline and during the follow-up period at 3 months (group 2), 6 months (groups 1 and 2) and 1 year (group 1 and 2). Tissue samples of the right and left atrial appendages in patients in group 1 are analysed for Heat Shock Protein levels and for tissue characteristics. Early post procedural atrial fibrillation is detected by continuous rhythm monitoring, whereas late post procedural atrial fibrillation is documented by either electrocardiogram or 24-h Holter registration.

**Conclusion:**

HALT & REVERSE aims to identify the correlation between Heat Shock Protein levels and degree of electropathology. The study outcome will contribute to novel diagnostic tools for the early recognition of clinical atrial fibrillation.

Trial Registrations: Rotterdam Medical Ethical Committee MEC-2014-393, Dutch Trial Registration NTR4658

## Background

Atrial fibrillation (AF) initially presents with short, self-terminating episodes and progresses into long-lasting episodes which are unlikely to convert spontaneously to sinus rhythm [1]. The exact mechanism underlying this progression is unknown. At present, there are no diagnostic tests or biomarkers available predicting recurrences of progressive AF episodes. In daily clinical practice, we can for example, not predict which patients develop AF following cardiac surgery or AF recurrences after pulmonary vein isolation (PVI) or electrical cardioversion (ECV).

AF persistence induces atrial remodeling on an electrical, structural and contractile level, also referred to as electropathology [2]. Electrical changes such as shortening of AF cycle length and contractile dysfunction occur within days after AF onset [3, 4]. After weeks of AF persistence, structural alterations become manifest in the extracellular matrix and in cardiomyocytes [1, 5–7]. Electrical and contractile abnormalities are completely reversible within weeks or days after restoration of sinus rhythm [3, 7–10], whereas recovery from structural remodeling is slow and takes up to months [11]. This finding indicates that structural remodeling underlies AF progression and recurrences. Therefore, research is directed at identifying key pathways involved in structural remodeling.

**Fig. 1:**
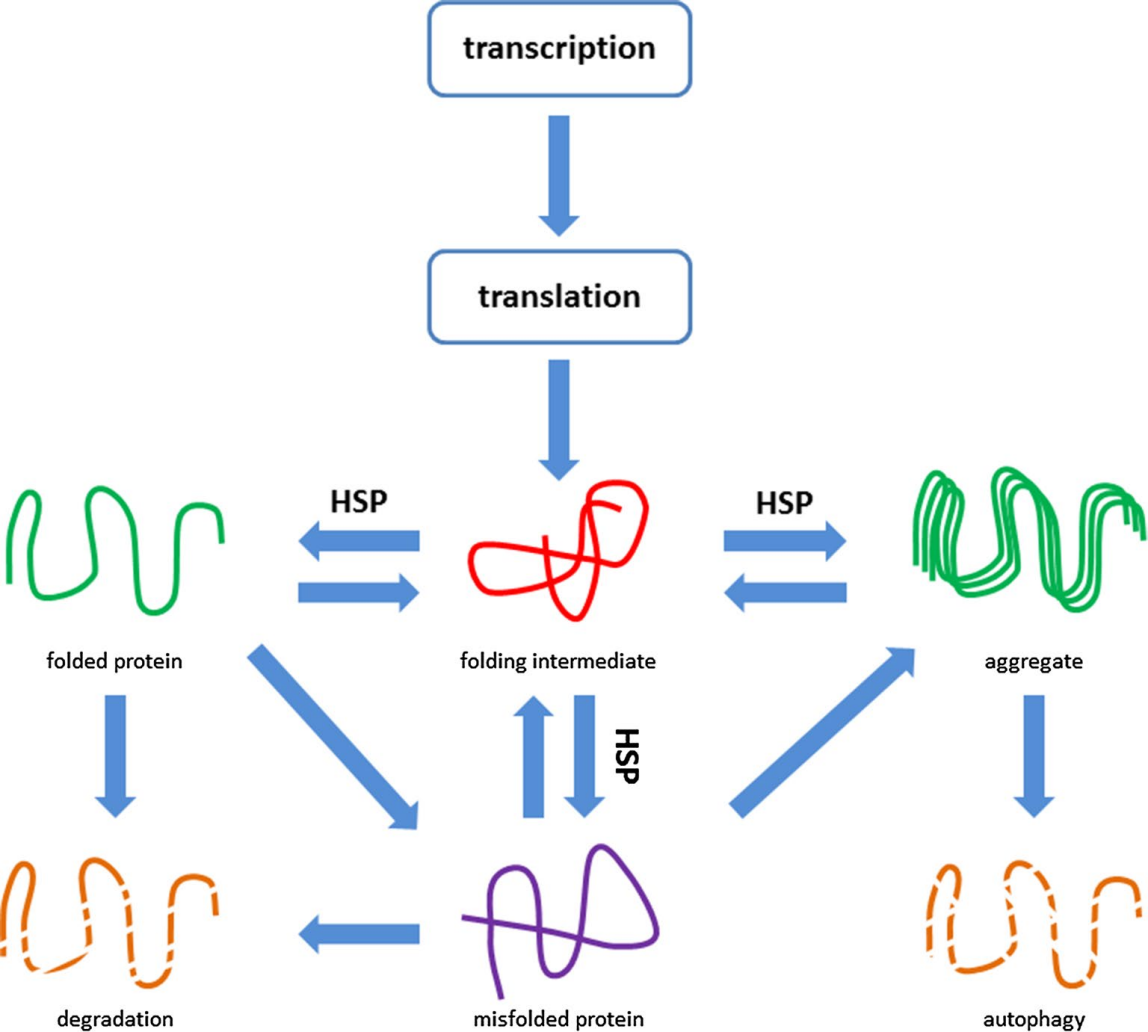
Proteostasis is the balance between protein expression, folding, function and clearance in the cell. When proteins are generated at the ribosomes after translation, chaperones, such as heat shock proteins, guide proper protein folding, and when not folded correctly, they guide remodeling of the protein. Furthermore, these chaperones have a role in limiting protein aggregation and they have a role in protein degradation

**Fig. 2:**
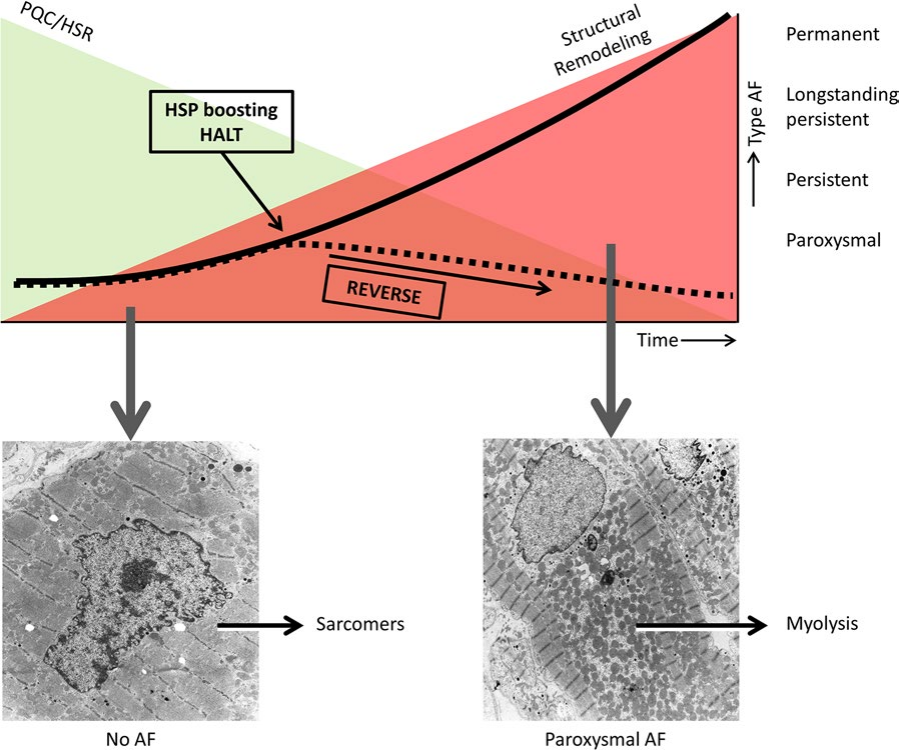
Concept of the HALT & REVERSE project. In the normal heart Protein Quality Control (PQC) and the Heat Shock Response (HSR, *upper panel*: *green area*) maintain proteostasis in times of stress. However, with the duration of AF and with increasing age, the heat shock protein (HSP) levels get exhausted. This leads to a derailment of proteostasis (*upper panel*: *red area*), resulting in structural remodeling and contractile dysfunction of cardiomyocytes, because of, amongst others, degradation of sarcomers (myolysis, *left* and *right lower panel*). AF progresses in time (*upper panel*: *solid line*), which is rooted in the underlying electropathology. By pharmacological boosting of HSPs, we aim to HALT or even REVERSE atrial electropathology and consequently AF progression (*upper panel*: *dashed line*)

Recently it was demonstrated that derailment of cardiomyocyte proteostasis, underlies AF progression and recurrences [12–15]. Cells, including cardiomyocytes, depend for proper functioning on the maintenance of proteostasis: the balance in protein synthesis, folding, assembling, trafficking, and clearance [16, 17] (Fig. 1). Chaperones, mainly consisting of Heat Shock Proteins (HSPs), assist in the maintenance of proteostasis [17–19]. Upon acute stress e.g. in paroxysmal AF, HSPs are upregulated [20]. The progressive nature of AF lies in the gradual, often age-related, exhaustion of the HSP levels and subsequent derailment of proteostasis, resulting in structural remodeling and contractile dysfunction of cardiomyocytes (Fig. 2). Therefore, compounds that boost HSP expression, such as geranylgeranylacetone (GGA) and GGA derivatives [21, 22], are of clinical interest. Indeed, HSPs boosting, via Heat Shock Factor-1 activation, protected against derailment of proteostasis and AF progression and recurrences in several experimental AF models, including tachypaced cardiomyocytes [23], *Drosophilas* [15] and canine models [24].

The HALT & REVERSE (*Hsf1ActivatorsLowerCardiomyocyt damage:Towards a novel approach toREVERSEatrial fibrillation*, MEC 2014-393, NTR 4658) project focusses on the correlation between HSPs and progression of electropathology [2]. Hereto, the HSP levels in blood and atrial tissue samples are determined and related to electrophysiological characteristics of the atria obtained from epicardial mapping during cardiothoracic surgery and endovascular mapping during PVI. The findings will elucidate whether HSP levels and electrophysiological characteristics represent a novel diagnostic tool (a so-called “bio-electrical fingerprint”) to predict the clinical outcome for AF patients and/or cardiac surgery.

## Study design

HALT & REVERSE is a prospective observational study, with a planned duration of 48 months. This study is carried out according to the principals of the Declaration of Helsinki Version and in accordance with the Medical Research involving Human Subjects Act. The study is approved by the Rotterdam local medical ethical committee (MEC 2014-393).

### Study objectives

The primary study objectives are to test the correlation between atrial electropathology as visualised by high-resolution epicardial mapping or endovascular mapping, HSP levels, atrial tissue characteristics and clinical characteristics in patients undergoing either open chest cardiac surgery, PVI or ECV. Secondly, the correlation between HSP levels and development or recurrence of AF in patients undergoing cardiothoracic surgery, PVI or ECV is tested.

### Study population

For the HALT & REVERSE project, three separate study groups are defined, consisting of patients scheduled for (1) cardiothoracic surgery, (2) PVI and (3) ECV for symptomatic AF. Each group contains 100 consecutive patients. Patients are recruited at the Department of Cardiothoracic Surgery or at the Department of Cardiology at the Erasmus Medical Center, Rotterdam, The Netherlands. Prior to enrolling in the study, each patient is provided an oral and a written explanation of the study procedure. Written informed consent is obtained from all patients.

**Fig. 3:**
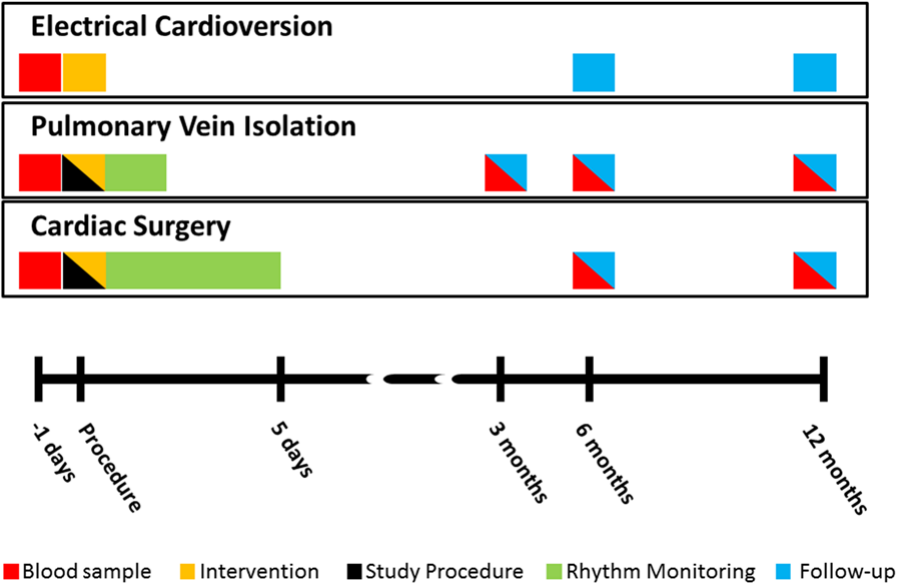
Timecourse of the HALT & REVERSE project. A baseline blood sample (*red bar*) is obtained from all patients 1 day prior to the procedure. In the pulmonary vein isolation—and cardiac surgery group, the study procedure (*black bar* epicardial or endocardial mapping procedure) is performed during the elective therapeutic intervention (*yellow bar*) and post-procedural continuous rhythm monitoring afterwards (*green bars*). The *blue bars* indicate long term follow-up (>3 months) including a visit to the outpatient clinic with electrocardiography, blood samples and 24-h Holter recordings when indicated

Blood samples for baseline HSP level determination taken from all patients prior to the scheduled intervention (surgery, PVI or ECV) (Fig. 3). Patient characteristics (e.g. age, medical history, cardiovascular risk factors) are obtained from the patient’s file.

### Inclusion criteria

In order to be eligible to participate in this study, all subjects must be >18 years old. In the cardiothoracic surgery group, only patients scheduled for elective cardiothoracic surgery for structural heart disease with or without a history of AF are included.

The PVI group will enrol patients with paroxysmal or (long-standing) persistent AF. The ECV group will consist of patients with symptomatic, AF presenting for electrical cardioversion.

### Exclusion criteria

A potential subject who meets any of the following criteria in all three study groups are excluded from participation in this study: paced atrial rhythm, haemodynamic instability, presence of cardiac assist devices, usage of inotropic agents prior to intervention or patients undergoing emergency or redo cardiac surgery.

### Sample size calculation

Approximately 25 % of patients undergoing cardiothoracic surgery develop early postoperative AF [25]. Estimated AF recurrence rate 1 year after PVI is 45 % as has been shown in previous studies [26–28], whereas the arrhythmia tends to recur within 1 year in 25–40 % [29–31] after ECV.

For none of our study groups, data testing the correlation between HSP and AF development or recurrence (in humans) is available. Therefore, a reliable parameter for sample size calculation is not available. Based upon the data mentioned above, we expect a pilot study with 100 patients in each group to be suitable for further determination of the exact number of patients needed to generate a power of 0.95 with a significance of 0.05.

### Intra-operative mapping procedure

**Fig. 4:**
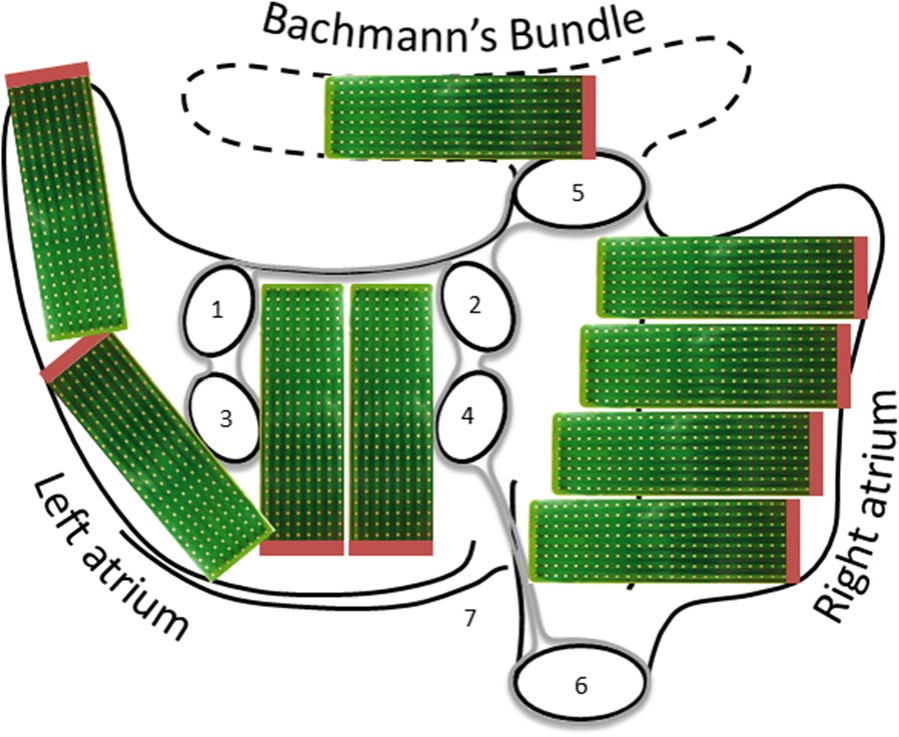
Epicardial mapping scheme. *1* Left superior pulmonary vein; *2* right superior pulmonary vein; *3* left inferior pulmonary vein; *4* right inferior pulmonary vein; *5* inferior caval vein; *6* superior caval vein; *7* coronary sinus. The left and right atrium and Bachmann’s Bundle are mapped following a predefined epicardial mapping scheme along anatomical structures, by the use of an 192 polar electrode array (*green*)

High-resolution epicardial mapping of the entire atria according to a previously described mapping scheme is performed during open chest cardiac surgery [32]. A reference electrode is temporarily attached to the right atrial appendage. An indifferent electrode consists of a steel wire, placed in subcutaneous tissue. The mapping procedure will be performed using a custom made 192-site electrode array [32] with a 2 mm inter-electrode distance. Recordings are made at nine consecutive sites following a predefined mapping scheme (Fig. 4) during sinus rhythm (5 s/site), during pacing maneuvers for inducing AF (1 site, Bachman’s Bundle), and during AF (10 s/site). Pacing maneuvers will be performed with epicardial atrial fixed rate pacing at 250–300–350 beats per minute successively, using a standard temporary pacemaker device. If AF sustains until the end of the mapping procedure, sinus rhythm will be restored with 5–10 Joule synchronous electric cardioversion.

After introduction cannulas of the cardiopulmonary bypass into the right atrium via the right atrial appendage (RAA) a tissue sample is obtained from the incision site in all patients. A tissue sample of the left atrial appendage (LAA) is obtained if the LAA is incised or amputated during surgery. All collected tissue samples are immediately snap-frozen in liquid nitrogen and stored at −80 °C.

After the surgical procedure, heart rhythm is continuously monitored until hospital discharge in order to detect early post-operative AF (PoAF). PoAF is defined as a complete irregular heartbeat without consistent P waves, sustaining for at least 30 s. At 6 months and 1 year after the procedure, patients visit the outpatient clinic for detection of late post-operative AF. A 12-lead electrocardiogram and a blood sample for HSP analysis are obtained. A 24-h Holter registration is performed when AF is suspected.

### Pulmonary vein isolation

An endovascular mapping procedure is performed prior to isolation of the pulmonary veins. Atrial electropathology is studied by means of programmed electrical stimulation in the right and the left atrium, including fixed rate atrial pacing, delivery of atrial extra systoles and decremental pacing maneuvers. Recordings of the right atrium are made by a decapolar catheter at the right atrial free wall and pacing is performed from the ablation catheter, which is positioned in the RAA. Mapping in the left atrium is performed by placing a Lasso catheter at the posterior wall and a decapolar catheter in the coronary sinus, while pacing from the left atrial roofline. Subsequently, the PVI is performed according to the electrophysiologist’ choice by either radiofrequency ablation or cryoballoon ablation.

According to standard care, heart rhythm is monitored continuously until discharge. Recordings are analyzed to detect early AF episodes, sustained for at least 30 s. At 3, 6 months and 1 year after the procedure, patients visit the outpatient clinic. During these visits, 12-lead ECGs and 24-h Holter registrations are performed to detect late post procedural AF recurrences and an additional blood sample is obtained for HSP level determination.

### Electrical cardioversion

Patients will be interviewed by telephone at 6 and 12 months to detect AF recurrences.

### Tissue analysis

All obtained blood samples and atrial tissue samples are stored at −80 °C until analysis. All samples are tested for HSP biomarkers, including HSP70 and HSP27. Biomarker levels are determined by commercially available ELISA’s and Western blot analysis.

## Main study parameters

### Intra-operative mapping

**Fig. 5:**
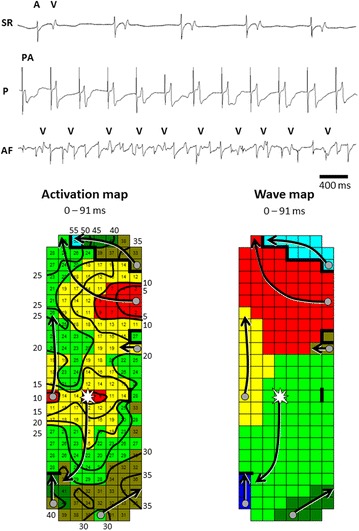
Epicardial high-resolution mapping of the atria. Atrial electrograms recorded during sinus rhythm (SR), atrial pacing (P) and (electrically induced) atrial fibrillation (AF) obtained during the epicardial mapping procedure are shown in the *upper panel*. The *left lower panel* shows an example of an activation map during electrically induced AF of the right atrium in a patient undergoing coronary artery bypass grafting without a history of AF. Isochrones are drawn at 5 ms, areas of conduction block are indicated by *black bars*, the origin of peripheral and epicardial breakthrough waves by respectively *grey dots* and *white asterisks*. The *arrows* indicate main activation direction. Corresponding wavemap is displayed in the *right lower panel*. The wavemap shows the different types of fibrillation waves entering the mapping area from various directions. These maps are used to determine the incidence of e.g. conduction block and epicardial breakthrough

Custom-made software used for off-line analysis of the recordings have previously been described in detail [32–34]. Local activation times of the unipolar atrial potentials are automatically detected in order to reconstruct color-coded activation-, conduction block-, breakthrough-, voltage- and wavemaps during sinus rhythm and AF [32–34]. An example of an activation and wavemap constructed during AF is given in Fig. 5. The maps are used to quantify electrophysiological variables. These variables are correlated with the pre-operative HSP level and development of post-operative AF.

### Pulmonary vein isolation

The degree of fractionated electrograms obtained from multiple sites in the right and left atrium are studied for fractionation and cycle length variation. HSP levels prior to PVI are correlated with the development of AF recurrence.

### Electrical cardioversion

Correlation between HSP levels prior to cardioversion and successful restoration of sinus rhythm and AF recurrences is determined.

### Study endpoints

For all groups, endpoint of the study is reached when AF develops after cardiac surgery or recurs after ECV or PVI. The correlation between several biomarkers (HSP70, HSP27) and development or recurrences of AF is determined, but also the correlation between degree of electropathology and development or recurrences of AF. The relationship between atrial electrophysiological variables, as visualised by epicardial mapping, and structural tissue characteristics are tested. The findings will indicate whether HSP levels and/or the degree of electropathology represent a novel diagnostic tool to predict the clinical outcome of AF progression and/or recurrences.

### Statistical analysis

The association between biomarker levels and development or recurrence of atrial fibrillation is calculated using multivariate logistic regression (for short term outcome) and cox regression models (for long-term outcome). Furthermore, Kaplan–Meier life tables are created for the three groups (cardiac surgery, PVI and ECV) and Log rank tests are used to compare the different groups.

ANOVA is utilized to compare various electrophysiological continuous parameters, for categorical parameters the Chi-square test will be used. Repeated HSP measurements are conducted by Joint modeling and mixed modeling analysis. Multiple biomarkers levels are corrected by ANOVA with Bonferoni adjustments.

## Conclusion

HALT & REVERSE aims to examine the interplay between electropathological substrate underlying AF, and HSP levels in the blood and atrial tissue, in order to predict development, recurrence or progression of AF. The results will elucidate whether HSP levels and electrophysiological characteristics can be used as a future diagnostic tool in the clinical setting.

## Abbreviations

AF: atrial fibrillation
ECG: electrocardiogram
ECV: electrical cardioversion
GGA: geranylgeranylacetone
HALT & REVERSE: Hsf1 activators lower cardiomyocyt damage; towards a novel approach to reverse atrial fibrillation
HSP: heat shock protein
LAA: left atrial appendage
PVI: pulmonary vein isolation
PoAF: post-operative atrial fibrillation
RAA: right atrial appendage
